# Epigenetic study of early breast cancer (EBC) based on DNA methylation and gene integration analysis

**DOI:** 10.1038/s41598-022-05486-3

**Published:** 2022-02-07

**Authors:** Wenshan Zhang, Haoqi Wang, Yixin Qi, Sainan Li, Cuizhi Geng

**Affiliations:** 1 Department of Breast Center, The Fourth Hospital of Hebei Medical University, 169 Tianshan Street, Shijiazhuang, Hebei 050011 People’s Republic of China; 2 Gland Surgery, Shijiazhuang People’s Hospital, Shijiazhuang, People’s Republic of China

**Keywords:** Breast cancer, Diagnostic markers

## Abstract

Breast cancer (BC) is one of the leading causes of cancer-related deaths in women. The purpose of this study is to identify key molecular markers related to the diagnosis and prognosis of early breast cancer (EBC). The data of mRNA, lncRNA and DNA methylation were downloaded from The Cancer Genome Atlas (TCGA) dataset for identification of differentially expressed mRNAs (DEmRNAs), differentially expressed lncRNAs (DElncRNAs) and DNA methylation analysis. Gene Ontology (GO) and Kyoto Encyclopedia of Genes and Genomes (KEGG) enrichment analyzes were used to identify the biological functions of DEmRNAs. The correlation analysis between DNA methylation and DEmRNAs was carried out. Then, diagnostic analysis and prognostic analysis of identified DEmRNAs and DElncRNAs were also performed in the TCGA database. Subsequently, methylation state verification for identified DEmRNAs was performed in the GSE32393 dataset. In addition, real-time polymerase chain reaction (RT-PCR) in vitro verification of genes was performed. Finally, AC093110.1 was overexpressed in human BC cell line MCF-7 to verify cell proliferation and migration. In this study, a total of 1633 DEmRNAs, 750 DElncRNAs and 8042 differentially methylated sites were obtained, respectively. In the Venn analysis, 11 keys DEmRNAs (ALDH1L1, SPTBN1, MRGPRF, CAV2, HSPB6, PITX1, WDR86, PENK, CACNA1H, ALDH1A2 and MME) were we found. ALDH1A2, ALDH1L1, HSPB6, MME, MRGPRF, PENK, PITX1, SPTBN1, WDR86 and CAV2 may be considered as potential diagnostic gene biomarkers in EBC. Strikingly, CAV2, MME, AC093110.1 and AC120498.6 were significantly actively correlated with survival. Methylation state of identified DEmRNAs in GSE32393 dataset was consistent with the result in TCGA. AC093110.1 can affect the proliferation and migration of MCF-7. ALDH1A2, ALDH1L1, HSPB6, MME, MRGPRF, PENK, PITX1, SPTBN1, WDR86 and CAV2 may be potential diagnostic gene biomarkers of EBC. Strikingly, CAV2, MME, AC093110.1 and AC120498.6 were significantly actively correlated with survival. The identification of these genes can help in the early diagnosis and treatment of EBC. In addition, AC093110.1 can regulate SPTBN1 expression and play an important role in cell proliferation and migration, which provides clues to clarify the regulatory mechanism of EBC.

## Introduction

Breast cancer (BC) is one of the three most common cancers in the world^1^. BC is the second leading cause of cancer-related death in women, and its morbidity and mortality are expected to increase significantly in the next few years^2,3^. Breast-conserving surgery and radiation therapy are used for the treatment of Stage I and II BC. Induction chemotherapy is usually required for stage III BC to shrink the tumor to promote breast-conserving surgery, or mastectomy in severe cases. Stage IV BC has a poor prognosis, and treatment options must strike a balance between prolonging life, reducing pain, and the harm caused by treatment^4^. It is noted that early breast cancer (EBC) is potentially curable^1^. Therefore, early detection and treatment are needed to reduce BC mortality.

Although the specific and complex pathological mechanism of BC is unclear, various genes have been reported to be involved in the pathogenesis of BC. Previous studies have found that Breast Cancer 1 protein (BRCA1) is a tumor suppressor, and decreased expression of BRCA1 disrupts breast differentiation and increases the risk of BC^5^. The partner and locator of BRCA2 (PALB2) is considered to be a BC susceptibility gene, and germline deletions in PALB2 lead to an increased risk of BC^6^. PICALM interacting mitotic regulator (PIMREG) is up-regulated in BC and positively correlated with clinical stage, lymph node metastasis type and poor survival. In addition, overexpression of PIMREG promotes BC invasiveness through constitutive activation of NF-κB signaling^7^. Surprisingly, long non-coding RNA (lncRNA) also plays an important regulatory role in BC^8^. High expression of lncRNA H19 can increase drug resistance in BC cells and is connected with poor prognosis in BC patients^9^. LncRNA small nucleolar RNA host gene 20 (SNHG20) may regulate human epidermal growth factor receptor 2 (HER2) through microRNA-495 (miR-495) and promote the proliferation, invasion and migration of BC cells^10^. Thus it can be seen that mRNAs and lncRNAs could play an important regulatory role in EBC.

Epigenetic changes in the tumor cell genome, such as DNA methylation, have significant effect in the formation of cancer^11^. The decreased expression of breast cancer metastasis suppressor gene 1 (BRMS1) in triple-negative breast cancer (TNBC) is related to DNA methylation modification, and demethylation can reactivate BRMS1 expression to inhibit cell invasion^12^. Progestin and adipoQ receptor family member 3 (PAQR3) is a tumor suppressor gene for BC. Studies have found that the down-regulation of PAQR3 expression in BC tissues is significantly related to the abnormal methylation of the gene promoter^13^. The discovery of methylation biomarkers was of great significance for the diagnosis and prognosis of cancer^14^. Based on previous studies, we speculate that DNA methylation plays an important role in the progress of EBC.

Although previous studies have shown that mRNA, lncRNA and DNA methylation play an important regulatory role in BC, we have not found integrated studies on DNA methylation with DEmRNA and DElncRNA. Key genes screened out through the integration of DNA methylation with DEmRNA and DElncRNA have more research significance for the diagnosis and treatment of EBC. In this study, high throughput data of EBC including mRNA, lncRNA and DNA methylation were deeply mined from The Cancer Genome Atlas data portal (TCGA). Finally, several candidate genes (ALDH1A2, ALDH1L1, HSPB6, MME, MRGPRF, PENK, PITX1, SPTBN1, WDR86, CAV2, AC093110.1 and AC120498.6) may be used as the diagnosis and treatment targets of EBC.

## Results

### Identification of differentially expressed mRNAs (DEmRNAs) and differentially expressed lncRNAs (DElncRNAs)

According to the screening criteria of false discovery rate (FDR) < 0.05 and absolute value of log2FoldChange > 2, 1633 DEmRNAs were obtained, including 1007 up-regulated and 626 down-regulated mRNAs. Top 10 up-regulated DEmRNAs were COL10A1, MMP11, NEK2, PPAPDC1A, COL11A1, PKMYT1, KIF4A, CST1, HSD17B6 and IBSP (Table 1). Inversely, top 10 down-regulated DEmRNAs were GPAM, TNS1, FHL1, LYVE1, MYOM1, CAV1, RDH5, NPR1, GYG2 and KCNIP2 (Table 1). The heat map of top100 DEmRNAs was shown in Supplementary Fig. 1A.

**Table 1:** Top 10 up/down-regulated DEmRNAs.

Symbol	Log2FoldChange	Pvalue	Padj	Up/Down
COL10A1	7.476532	5.72E-267	1.00E-262	Up
MMP11	6.301695	9.30E-230	8.17E-226	Up
NEK2	4.15731	2.17E-155	9.55E-152	Up
PPAPDC1A	5.264656	6.79E-140	1.33E-136	Up
COL11A1	6.040087	6.57E-139	1.15E-135	Up
PKMYT1	3.971369	2.78E-131	3.62E-128	Up
KIF4A	3.749621	3.72E-128	3.84E-125	Up
CST1	7.878195	2.21E-120	1.62E-117	Up
HSD17B6	3.051248	6.30E-120	4.43E-117	Up
IBSP	7.230393	8.54E-120	5.77E-117	Up
GPAM	-4.43081	2.04E-189	1.20E-185	Down
TNS1	-3.17294	1.72E-149	6.05E-146	Down
FHL1	-4.75573	6.88E-148	2.01E-144	Down
LYVE1	-5.16065	7.12E-145	1.79E-141	Down
MYOM1	-3.61935	4.13E-140	9.07E-137	Down
CAV1	-3.43951	1.88E-132	3.00E-129	Down
RDH5	-4.35398	1.17E-131	1.71E-128	Down
NPR1	-3.74726	2.89E-131	3.62E-128	Down
GYG2	-4.29206	8.26E-131	9.68E-128	Down
KCNIP2	-5.23797	6.27E-129	6.88E-126	Down

According to the screening criteria of FDR < 0.05 and absolute value of log2FoldChange > 2, a total of 750 DElncRNAs were obtained, including 505 up-regulated and 245 down-regulated lncRNAs. Top 10 up-regulated DElncRNAs were LINC01614, LINC00922, LINC01705, LINC02544, AC134312.5, C6orf99, FAM83H-AS1, LINC01561, FOXD3-AS1 and AC015849.5 (Table 2). Inversely, top 10 down-regulated DElncRNAs were LINC01537, LINC02202, ENSG00000275149, AP001528.2, AC100771.2, AL139260.1, AL445426.1, AL031316.1, TRHDE-AS1 and AC048382.5 (Table 2). The heat map of top100 DElncRNAs was shown in Supplementary Fig. 1B.

**Table 2:** Top 10 up/ down-regulated DElncRNAs.

ID	Symbol	Log2FoldChange	Pvalue	Padj	Up/Down
ENSG00000230838	LINC01614	5.9137901	7.01E-155	5.60E-151	Up
ENSG00000261742	LINC00922	4.581607	1.42E-84	2.27E-81	Up
ENSG00000232679	LINC01705	5.793776	9.16E-80	1.05E-76	Up
ENSG00000261039	LINC02544	3.8234215	1.60E-78	1.59E-75	Up
ENSG00000261327	AC134312.5	3.4911273	1.24E-72	1.10E-69	Up
ENSG00000203711	C6orf99	2.7644663	2.17E-67	1.44E-64	Up
ENSG00000282685	FAM83H-AS1	2.3663007	1.24E-66	7.07E-64	Up
ENSG00000177234	LINC01561	5.2611068	3.18E-66	1.59E-63	Up
ENSG00000230798	FOXD3-AS1	4.8415738	5.97E-64	2.65E-61	Up
ENSG00000270977	AC015849.5	3.8331805	2.30E-63	9.20E-61	Up
ENSG00000227467	LINC01537	-3.716398	1.60E-92	6.37E-89	Down
ENSG00000245812	LINC02202	-3.165369	2.81E-92	7.47E-89	Down
ENSG00000275149	ENSG00000275149	-4.014873	3.56E-90	7.11E-87	Down
ENSG00000255471	AP001528.2	-3.191604	5.47E-83	7.28E-80	Down
ENSG00000254862	AC100771.2	-2.933096	4.65E-70	3.71E-67	Down
ENSG00000228436	AL139260.1	-2.004837	7.50E-69	5.44E-66	Down
ENSG00000231246	AL445426.1	-3.754067	1.00E-66	6.16E-64	Down
ENSG00000227591	AL031316.1	-4.281639	2.89E-66	1.54E-63	Down
ENSG00000236333	TRHDE-AS1	-5.573932	3.31E-65	1.56E-62	Down
ENSG00000275120	AC048382.5	-2.021649	7.68E-64	3.23E-61	Down

### Enrichment analysis of DEmRNAs

**Figure 1:**
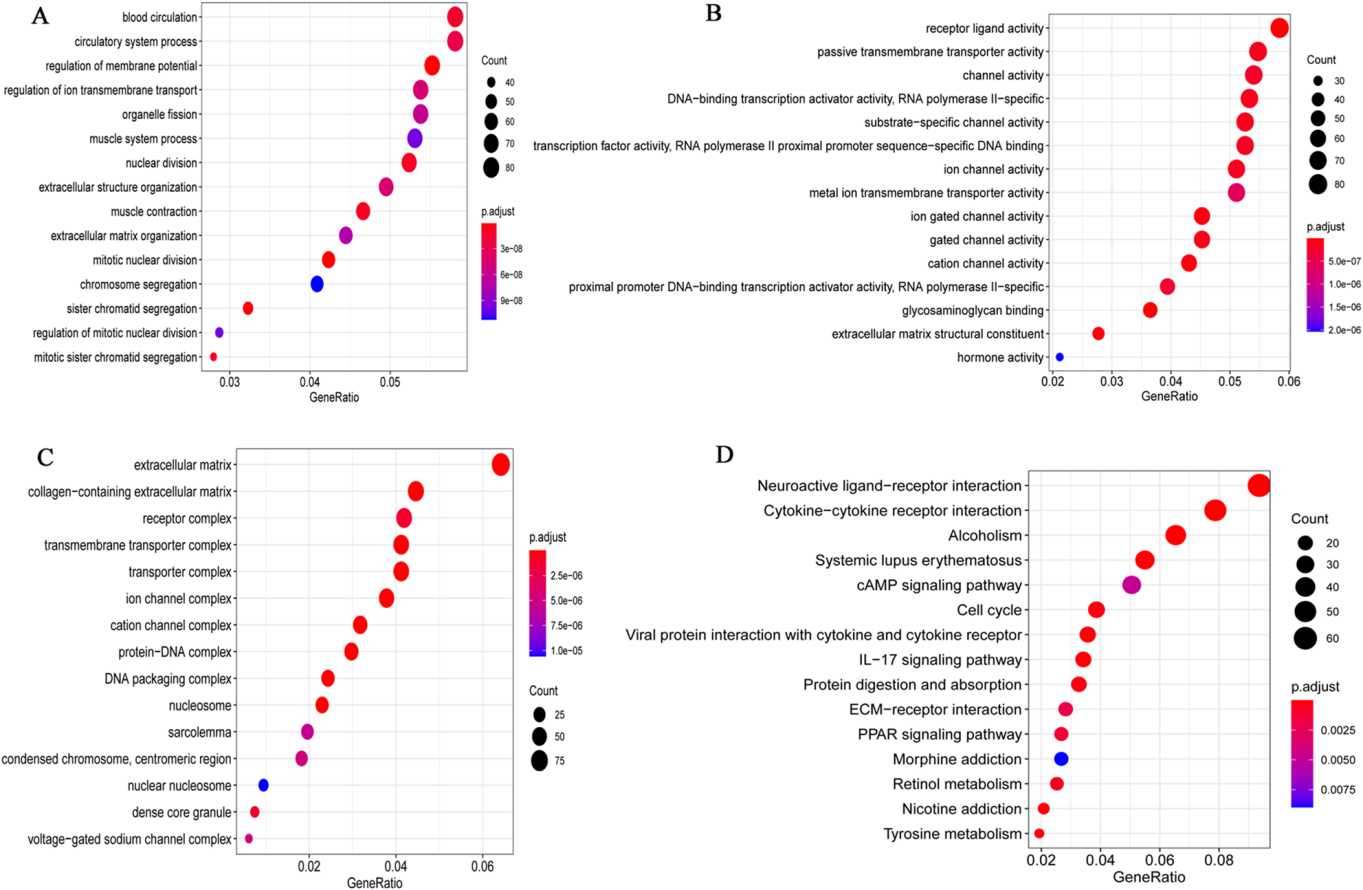
Top 15 significantly enriched GO terms and KEGG pathways of all DEmRNAs. (**A)** Biological process (BP); **(B)** Molecular function (MF)**; (C)** Cell composition (CC)**; (D)** Kyoto Encyclopedia of Genes and Genomes (KEGG) pathways. The x-axis and y-axis represent Gene Ratio and GO terms or KEGG pathways, respectively. The size of the dot represents the number of genes. The color of the dot represents the level of P value.

In order to explore the biological function of DEmRNAs, Gene Ontology (GO) and Kyoto Encyclopedia of Genes and Genomes (KEGG) functional enrichment were performed using clusterProfiler (version 3.10.1). FDR < 0.05 was considered statistically significant. In terms of biological process (BP), DEmRNAs were involved in extracellular matrix organization, mitotic nuclear division and chromosome segregation (Fig. 1A). In terms of molecular function (MF), DEmRNAs were involved in channel activity, DNA-binding transcription activator activity, RNA polymerase II-specific and transcription factor activity, RNA polymerase II proximal promoter sequence-specific DNA binding (Fig. 1B). In terms of cell composition (CC), DEmRNAs were involved in extracellular matrix, receptor complex and transmembrane transporter complex (Fig. 1C). According to KEGG enrichment analysis, neuroactive ligand-receptor interaction, cAMP signaling pathway and cell cycle were significantly enriched signaling pathways (Fig. 1D). From the functional enrichment, we found that most DEmRNA were involved in cell activity, cell cycle, transcription and so on. These biological processes may be related to the occurrence and development of BC.

### DNA methylation analysis

**Figure 2:**
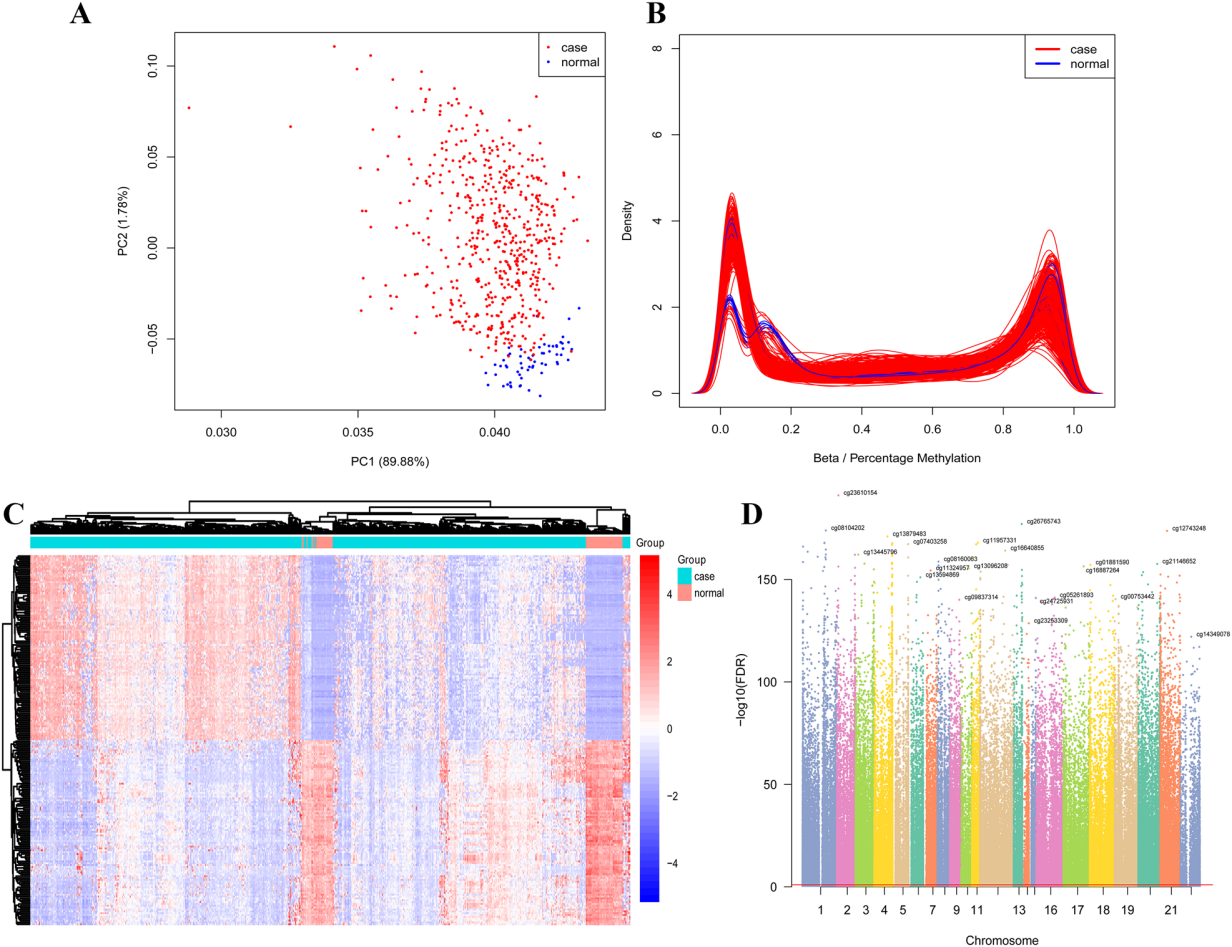
Analysis of differential methylation sites. (**A**) PCA of methylation sites; (**B**) The distribution of the β value of the sample; (**C**) Heat map of top 200 DMSs. Complete‑linkage method combined with Euclidean distance is used to construct clustering. Each row represents DMSs, and each column represents a sample. DMSs clustering tree is shown on the left. Red indicates above the reference channel (high expression genes). Blue indicates below the reference channel (low expression genes); (**D**) The Manhattan figure of DMSs in chromosome. The x-axis and y-axis represents the chromosomeand and the -log10 (FDR) of DMSs, respectively.

**Figure 3:**
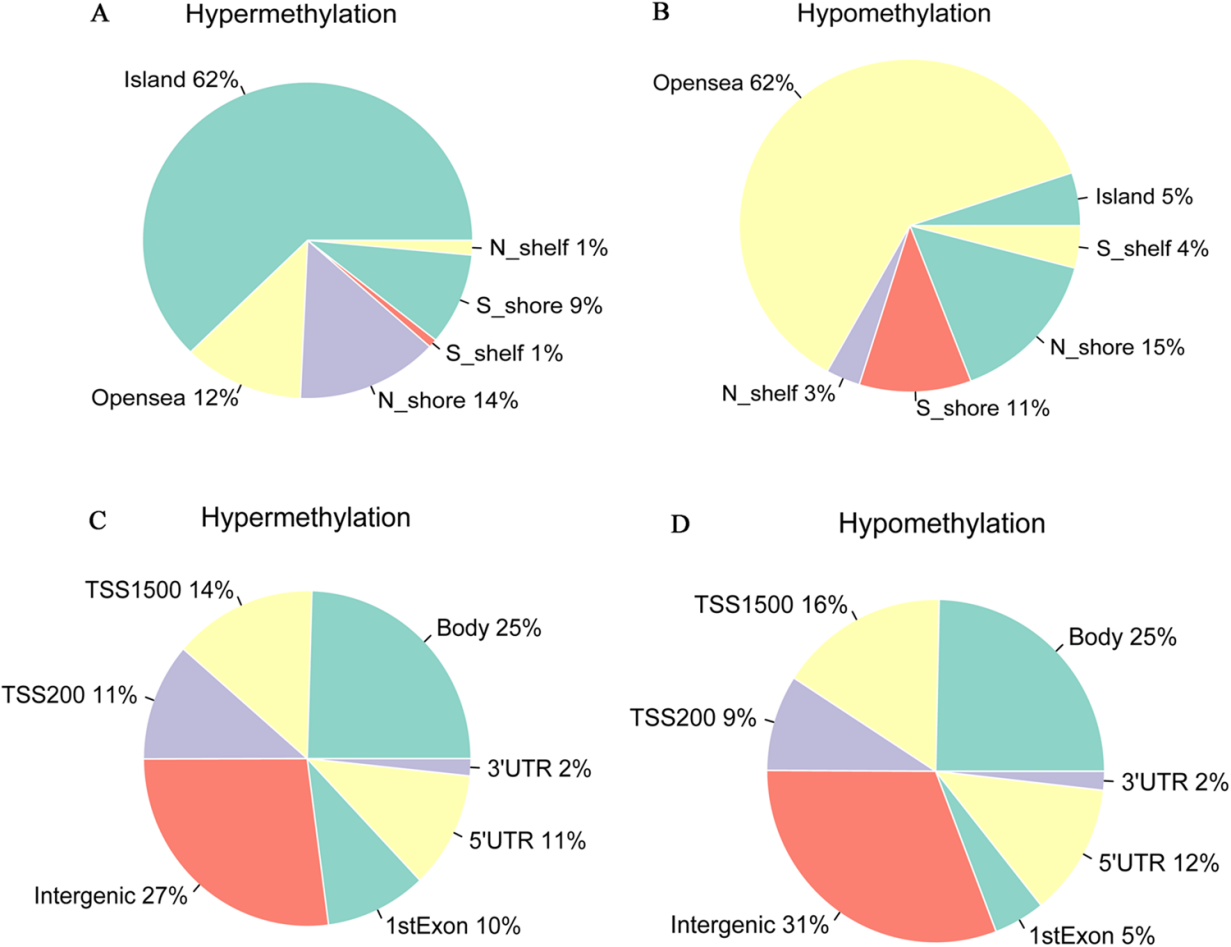
The distribution of differential methylation sites in CpG_Island and Gene_Group. (**A**) Distribution of hypermethylation sites on CpG_Island; (**B**) Distribution of hypomethylation sites on CpG_Island; (**C**) Distribution of hypermethylation sites on Gene_Group; (**D**) Distribution of hypomethylation sites on Gene_Group.

A total of 485,577 methylation sites were detected in this study. In order to ensure the reliability of the difference results, these 485,577 methylation sites were preprocessed to exclude unqualified methylation sites. After preprocess, 385,717 methylation sites were obtained and principal component analysis (PCA) was performed (Fig. 2A). Then, β > 0.7 or β < 0.3 were selected for methylation sites. (Fig. 2B). According to screening criteria of Δβ > 0.2 and FDR < 0.05, 8042 differentially methylation sites (DMSs) (7458 hypermethylation sites and 584 hypomethylation sites) were obtained. The heat map of top 200 DMSs was shown in Fig. 2C. The Manhattan figure of these DMSs was shown in Fig. 2D. The distribution of DMSs on CpG island and gene group was shown in Fig. 3. In addition, according to screening criteria of Δβ > 0.2 and FDR < 0.05, 775 differentially methylated CpG islands (773 hypermethylation regions and 2 hypomethylation regions) were also obtained.

### Correlation analysis of DNA methylation with DEmRNAs

In order to obtain DEmRNAs adjacent to DElncRNAs, DEmRNAs within 100 kb upstream and downstream of DElncRNAs were searched. A total of 249 pairs of DElncRNAs-adjacent DEmRNAs were obtained (including 197 DElncRNAs and 202 DEmRNAs). Then, the intersection of DElncRNAs cis regulated DEmRNAs and mRNAs corresponding to the DMSs was identified. A total of 50 DEmRNAs (marked as site & cis target) jointly regulated by DElncRNAs and DNA methylation were obtained. According to Pearson correlation analysis (absolute value of cor > 0.2, *P* < 0.05), a total of 458 pairs of DMSs-DEmRNAs (including 188 DEmRNAs and 452 DMSs) were obtained (Table S1). Among them, 109 positive correlation pairs and 349 negative correlation pairs. Subsequently, 311 relationship pairs were selected according to hypermethylation site-DEmRNAs with low expression or hypomethylation site-DEmRNAs with high expression. Among which, 127 DEmRNAs (marked as cor-relation) were included. In addition, the relationship between DEmRNAs and differential methylated genes on CpG islands was integrated. A total of 42 DMRs-DEmRNAs were identified (Table S2), which includes 42 pairs of hypermethylation with low expression DEmRNAs (marked as island) and 0 pairs of hypomethylation with highly expression DEmRNAs.

### Intersection analysis of DEmRNAs

**Figure 4:**
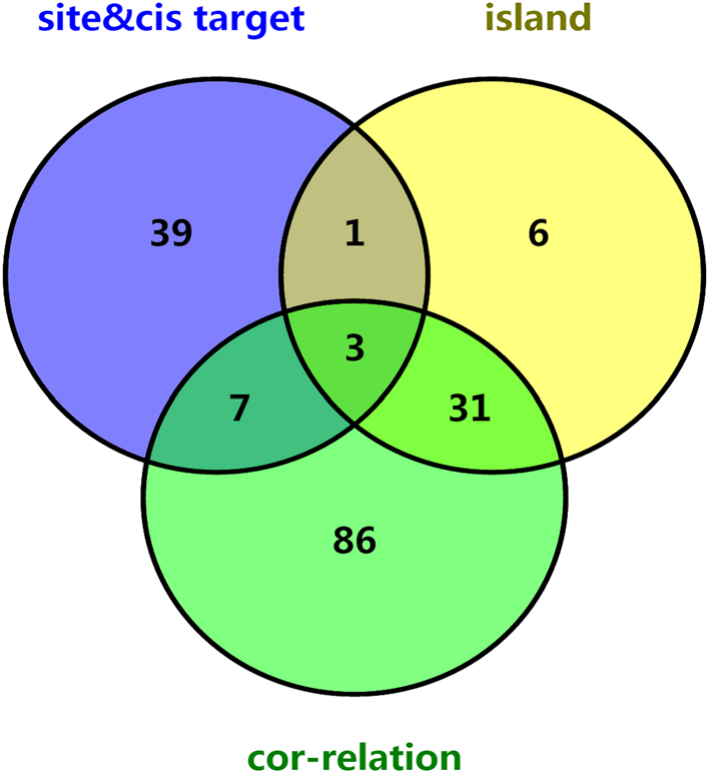
Venn diagram for mRNAs of site&cis target, cor-relation and island. Purple represent DEmRNAs co-regulated by DElncRNAs and DNA methylation. Green represent DEmRNAs negatively associated with DMSs. Yellow represent DEmRNAs negatively associated with DMRs. 11 DEmRNAs that interacted with site&cis target were selected.

Venn analysis was performed on the DEmRNAs obtained from the site & cis target, cor-relation and island (Fig. 4). A total of 11 DEmRNAs (ALDH1A2, ALDH1L1, HSPB6, MME, MRGPRF, PENK, PITX1, SPTBN1, WDR86, CAV2 and CACNA1H) that interacted with site&cis target and the DElncRNAs involved in their cis regulation were selected for following studies (Table 3).

**Table 3:** 11 DEmRNAs that overlap with site&cis target and DElncRNA involved in its cis regulation.

ID	DElncRNA			DEmRNA		
	Symbol	P-value	Up/down	Symbol	P-value	Up/down
ENSG00000246022	ALDH1L1-AS2	1.10E-55	Down	ALDH1L1	7.24E-77	Down
ENSG00000238018	AC093110.1	7.69E-47	Down	SPTBN1	4.51E-116	Down
ENSG00000256508	MRGPRF-AS1	1.32E-45	Down	MRGPRF	4.71E-40	Down
ENSG00000237813	AC002066.1	1.12E-40	Down	CAV2	4.34E-97	Down
ENSG00000261625	AP003071.4	2.76E-39	Down	MRGPRF	4.71E-40	Down
ENSG00000267328	AC002398.2	2.89E-37	Down	HSPB6	3.19E-101	Down
ENSG00000277619	AC008406.3	1.07E-27	Up	PITX1	2.07E-71	Up
ENSG00000243836	WDR86-AS1	3.55E-24	Down	WDR86	2.43E-34	Down
ENSG00000254254	AC012349.1	4.72E-15	Down	PENK	8.29E-33	Down
ENSG00000261713	SSTR5-AS1	9.53E-15	Up	CACNA1H	3.86E-21	Up
ENSG00000259285	AC025431.1	2.66E-13	Down	ALDH1A2	1.98E-48	Down
ENSG00000243953	AC073359.1	5.12E-11	Down	MME	5.12E-57	Down
ENSG00000260403	AC120498.3	1.28E-08	Up	CACNA1H	3.86E-21	Up
ENSG00000261294	AC120498.6	1.08E-07	Up	CACNA1H	3.86E-21	Up
ENSG00000259910	AC120498.1	2.91E-07	Up	CACNA1H	3.86E-21	Up
ENSG00000260532	AL031598.1	3.13E-05	Up	CACNA1H	3.86E-21	Up

### Diagnostic analysis, prognostic analysis and methylation verification of DEmRNAs and DElncRNAs

**Figure 5:**
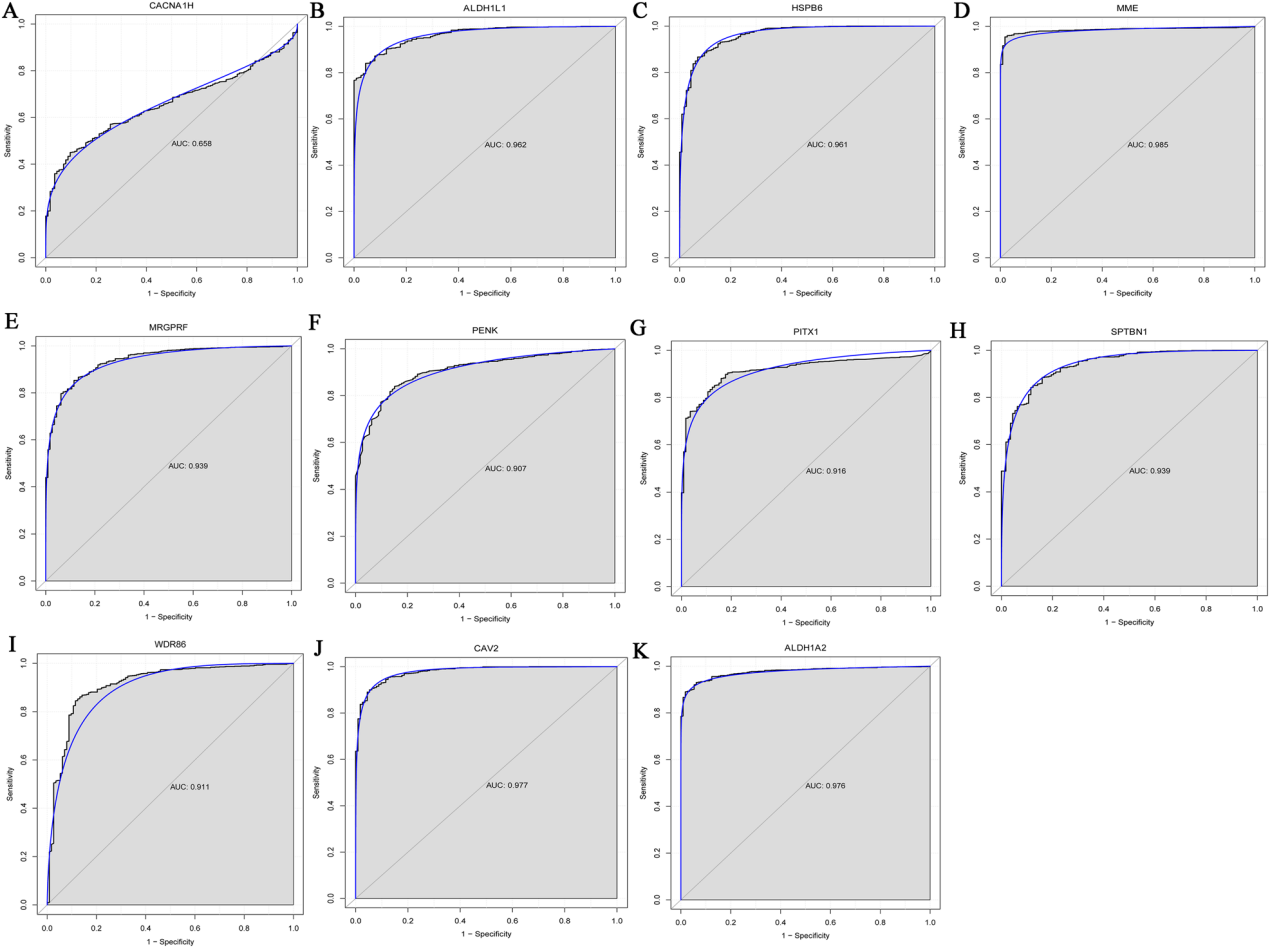
ROC curve of 11 diagnostic gene biomarkers. ROC curves were used to show the diagnostic ability with 1‑specificity and sensitivity. AUC > 0.9 represent a higher diagnostic value. AUC: area under curve, ROC: receiver operating characteristic.

**Figure 6:**
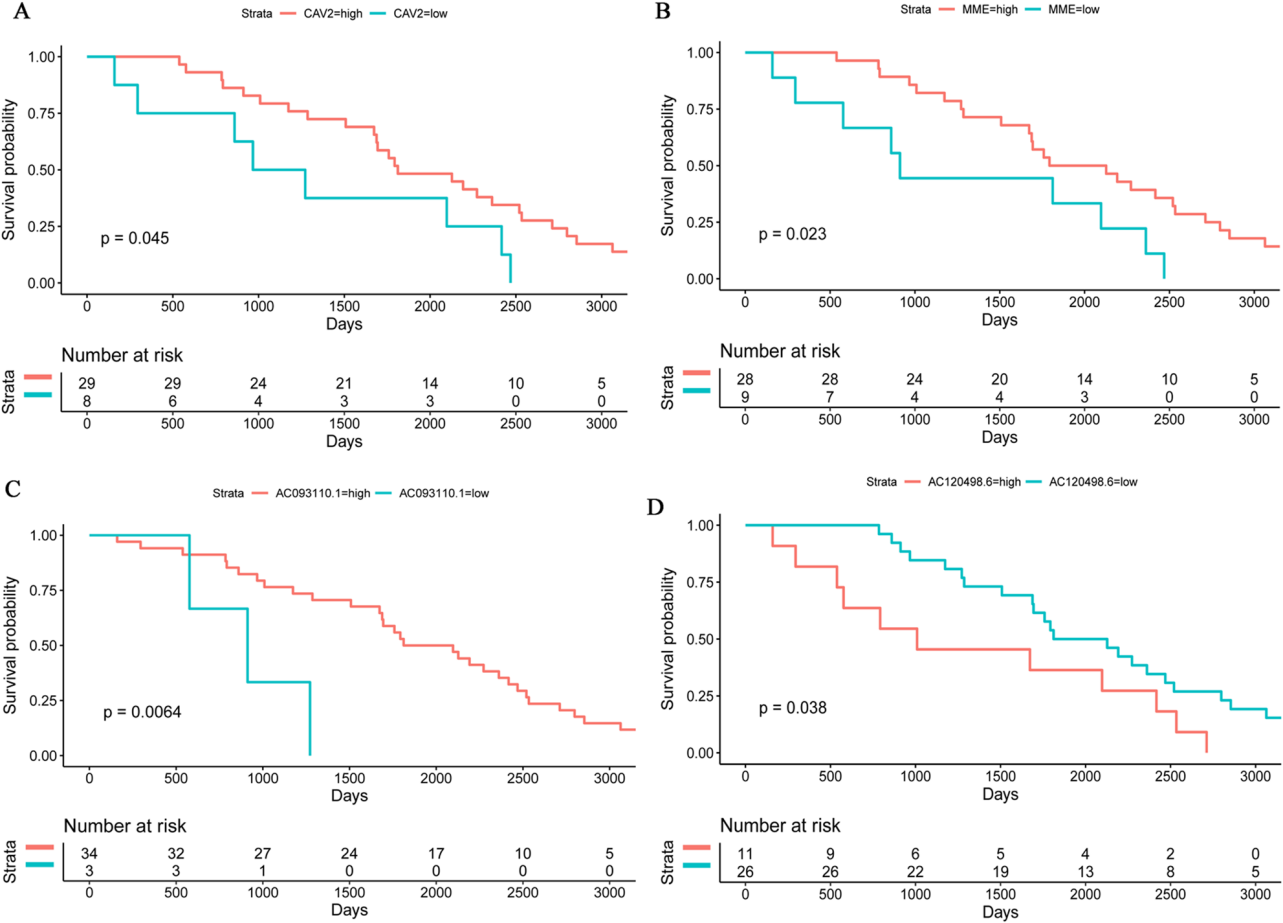
Prognostic analysis of CAV2, MME, AC093110.1 and AC120498.6 in TCGA database. The x-axis and y-axis represent time and survival probability, respectively. P < 0.05 was considered statistically significant.

**Figure 7:**
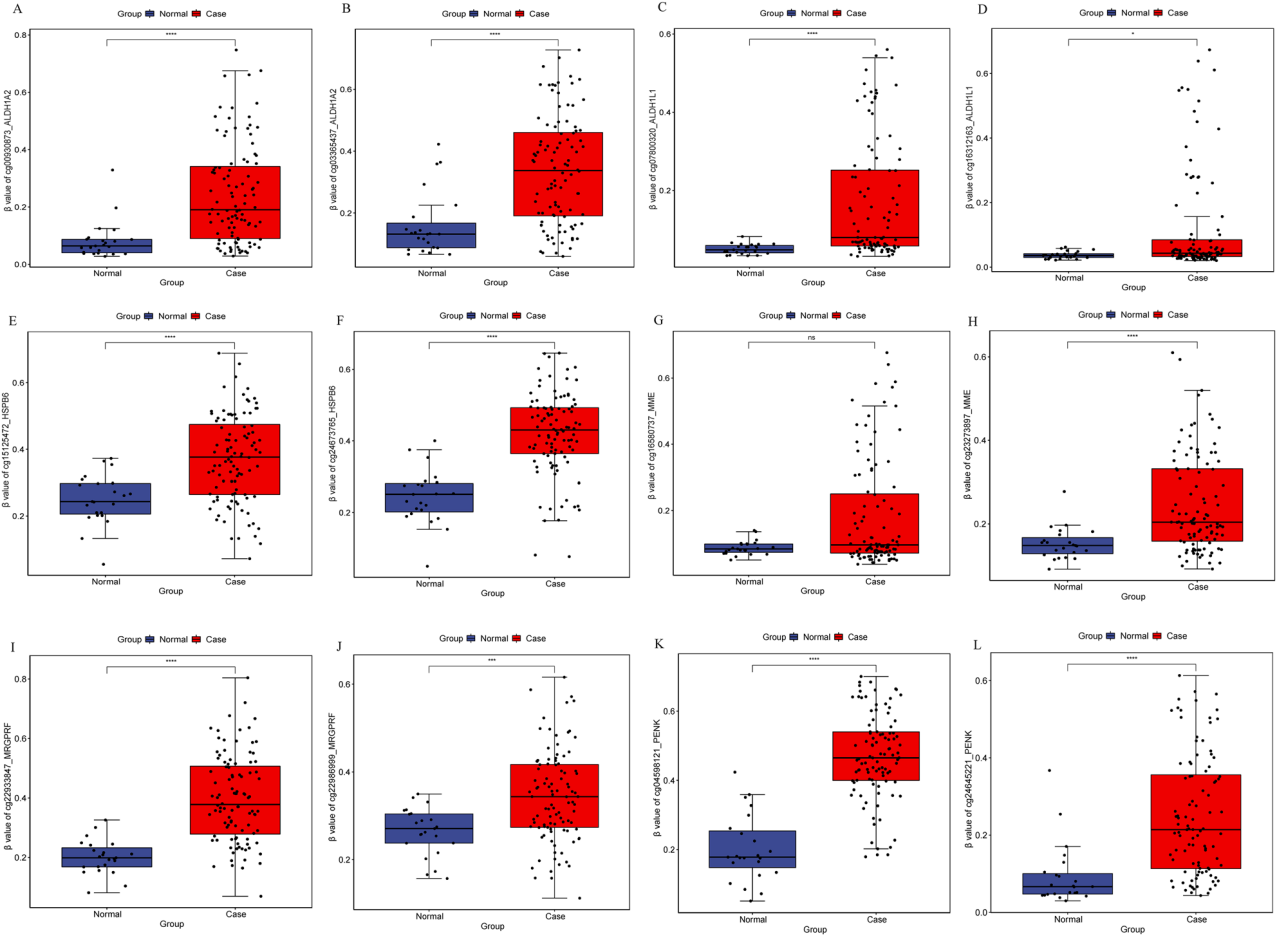
Methylation modification state verification of ALDH1A2, ALDH1L1, HSPB6, MME, MRGPRF and PENK in the GSE32393 dataset. * represents P < 0.05, ** represents P < 0.01, *** represents P < 0.001, P < 0.05 was considered statistically significant.

**Figure 8:**
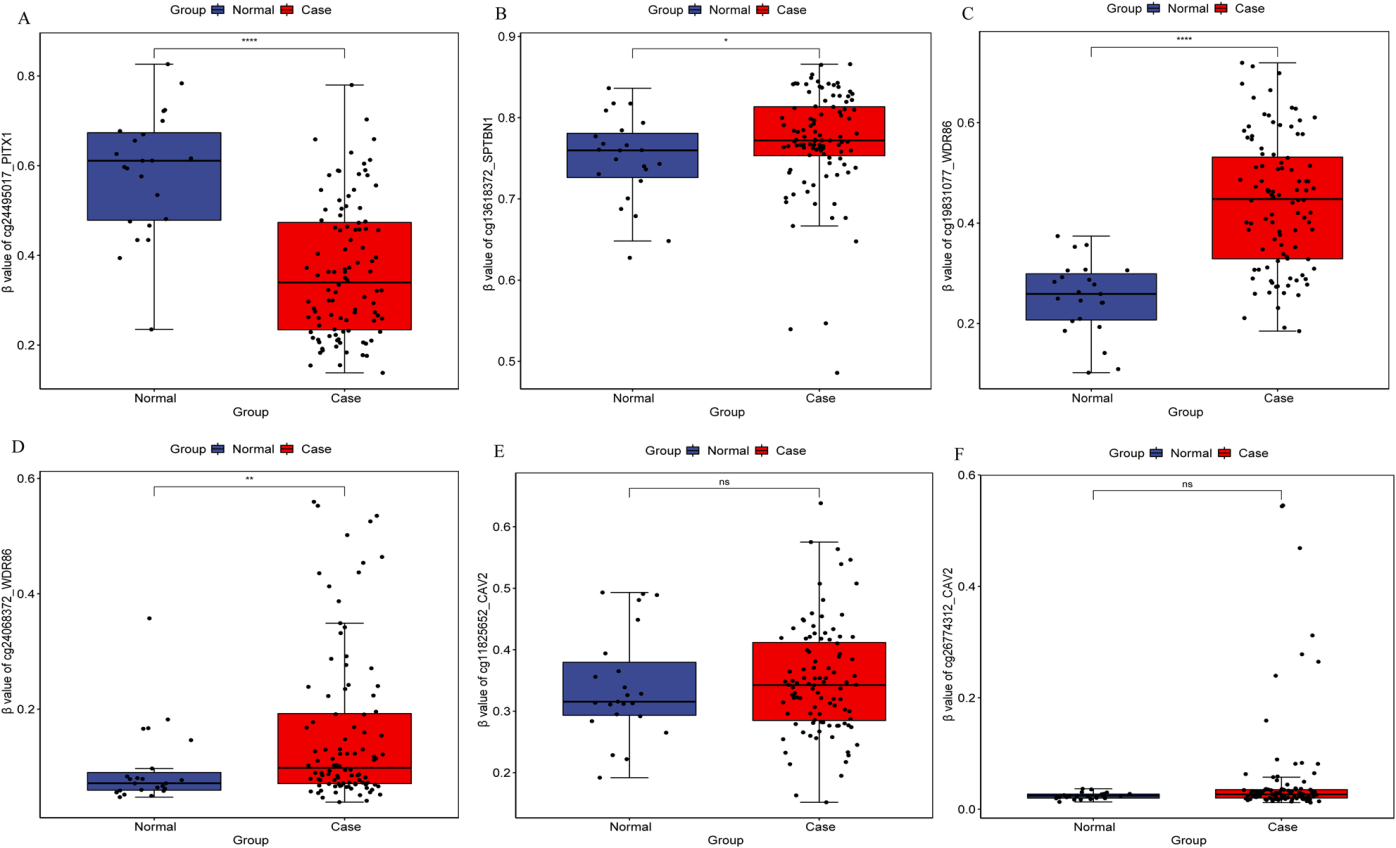
Methylation modification state verification of PITX1, SPTBN1, WDR86 and CAV2 in the GSE32393 dataset. * represents P < 0.05, ** represents P < 0.01, *** represents P < 0.001, P < 0.05 was considered statistically significant.

Diagnostic analysis of 11 DEmRNAs was performed (Fig. 5). In ROC analysis, the greater the AUC, the higher the diagnostic accuracy^15^. In receiver operating characteristic (ROC) curve analysis, the area under curve (AUC) of CACNA1H was 0.658 (Fig. 5A), which was not suitable as a diagnostic marker. Remarkably, the AUC of other DEmRNAs were all greater than 0.9 (Fig. 5B–K). The result showed that ALDH1A2 (hypermethylation), ALDH1L1 (hypermethylation), HSPB6 (hypermethylation), MME (hypermethylation), MRGPRF (hypermethylation), PENK (hypermethylation), PITX1 (hypomethylation), SPTBN1 (hypermethylation), WDR86 (hypermethylation) and CAV2 (hypermethylation) may be considered as the potential diagnostic gene biomarkers in EBC. Then, the prognosis analysis of 11 DEmRNAs and the DElncRNAs involved in their cis regulation was performed. The results showed that only CAV2, MME, AC093110.1 and AC120498.6 had prognostic value (Fig. 6), which indicated that these molecules were significantly actively correlated with survival. Subsequently, methylation modification state verification of ALDH1A2, ALDH1L1, HSPB6, MME, MRGPRF, PENK, PITX1, SPTBN1, WDR86 and CAV2 was performed in the GSE32393 dataset. The results showed that the methylation modification state was consistent with the result in TCGA (Figs. 7, 8).

### In vitro verification

**Figure 9:**
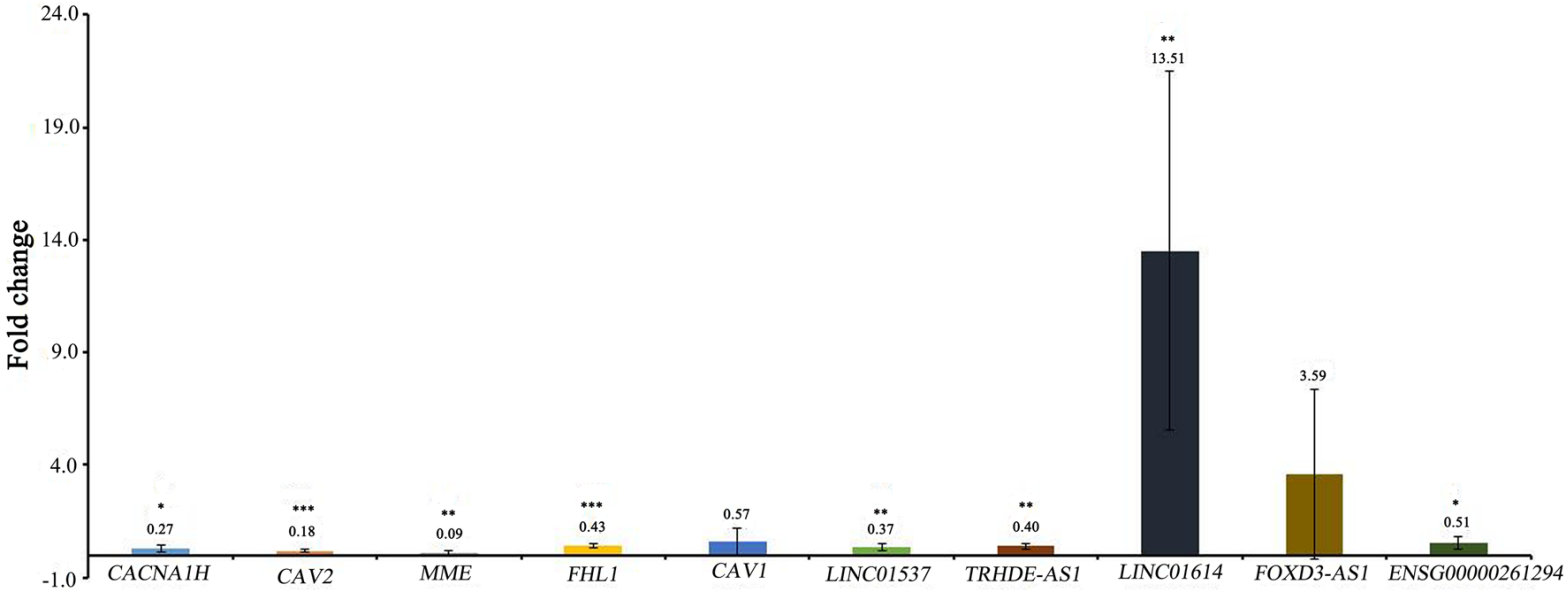
RT-PCR validation of CACNA1H, CAV2, MME, FHL1, CAV1, LINC01537, TRHDE-AS1, LINC01614, FOXD3-AS1 and ENSG00000261294 in tissues samples. * represents P < 0.05, ** represents P < 0.01, *** represents P < 0.001, Fold change > 1 represent regulation, Fold change < 1 represent regulation.

Clinical information of 6 EBC patients was shown in Table 4. The EBC tissue samples (disease group) and adjacent normal tissues samples (control group) were obtained for RT-PCR. Some top-ranked or reported genes, such as CACNA1H, CAV2, MME, FHL1, CAV1, LINC01537, TRHDE-AS1, LINC01614, FOXD3-AS1 and AC120498.6 (ENSG00000261294) were selected for RT-PCR verification. Primers were shown in Table 5. Compared with adjacent normal tissues, CAV2, MME, FHL1, CAV1, LINC01537, TRHDE-AS1 were down-regulated and LINC01614, FOXD3-AS1 were up-regulated in EBC tissues (Fig. 9), which was consistent with bioinformatics analysis. Moreover, except CAV1 and TRHDE-AS1, the expression of them the rest genes were significant (P < 0.05). However, CACNA1H and ENSG00000261294 were opposite with bioinformatics analysis. The inconsistency may be caused by the small sample size, which needs further study.

**Table 4:** Clinical information of patients in the RT-PCR.

Number	Age	Menstrual status	Tumor location	Pathological type	Tumor size	Clinical stages	Estrogen receptor (ER)	Progesterone receptor (PR)	Antigen KI67 (KI-67)	Human epidermal growth factor receptor-2 (HER-2)	Lymphatic metastasis	Histological grade
1	61	Postmenopause	Inner upper left breast	Invasive ductal carcinoma of the breast	3.5*3*3	IIA	Negative	Negative	50%	Positive, 3 +	0/13	Grade III
2	45	Premenopausal	External upper right breast	Invasive ductal carcinoma of the breast	2*1.5*1	IIA	Positive	Positive	30%	Negative	4/15	Grade I
3	57	Postmenopause	Behind the right nipple	Invasive ductal carcinoma	2.5*2.0*1.5	IIA	Positive	Positive	40%	Positive, 3 +	0/12	Grade III
4	51	Postmenopause	External inferior right breast	Invasive ductal carcinoma	1.5*1.5*1	I	Positive	Positive	30%	Positive, 3 +	1/33	Grade III
5	60	Postmenopause	External inferior right breast	Invasive ductal carcinoma	2.3*1.8*1.8	IIA	Positive	Positive	70%	Positive, 3 +	0/3	Grade III
6	51	Postmenopause	Under the areola above right nipple	Invasive ductal carcinoma	2.5*1.5*1.0	IIA	Positive	Positive	30%	Positive, 2 +	1/27	Grade III

**Table 5:** Primer sequence in the RT-PCR.

Primer name	Primer sequence (5' to 3')
GAPDH-F(internal reference)	5-CTGGGCTACACTGAGCACC-3
GAPDH-R(internal reference)	5-AAGTGGTCGTTGAGGGCAATG-3
ACTB-F(internal reference)	5-GATCAAGATCATTGCTCCTCCT-3
ACTB-R(internal reference)	5-TACTCCTGCTTGCTGATCCA-3
CACNA1H-F	5-ATGCTGGTAATCATGCTCAACTG-3
CACNA1H-R	5-AAAAGGCGAAAATGAAGGCGT-3
CAV2-F	5-ACGCGCATCAGTTCCCAAG-3
CAV2-R	5-TACCCGCCTCCACACTCAG-3
MME-F	5-GATCGCACTCTATGCAACCTAC-3
MME-R	5-TGTTTTGGATCAGTCGAGCAG-3
FHL1-F	5-GACTGGTCTAGGTGCTGCTC-3
FHL1-R	5-CATTTCAGGCAGCAGTGGTG-3
Cav1-F	5-GCCGCGTCTACTCCATCTAC-3
Cav1-R	5-CTGATGCGGATGTTGCTGAATA-3
LINC01537-F	5-ATCCACCCCTCAGTCCCAC-3
LINC01537-R	5-GGTGAGGTGAGGCAGGTCT-3
TRHDE-AS1-F	5-TCCCACTGAGTCTGCCCAC-3
TRHDE-AS1-R	5-GCTGCAGGGTGTATGGCTC-3
LINC01614-F	5-GGGACTTCAGACACGGAGAA-3
LINC01614-R	5-GGACACAGACCCTAGCACTT-3
FOXD3-AS1-F	5-ACACGGAACCCAATCCCTG-3
FOXD3-AS1-R	5-GAGGGAATCAGAAGCACCACT-3
ENSG00000261294-F	5-CTGGGACACCTGCCTCATTC-3
ENSG00000261294-R	5-CTCCAACTGGGGTGTTCTGAG-3

### AC093110.1 overexpression promoted proliferation and migration of MCF-7

**Figure 10:**
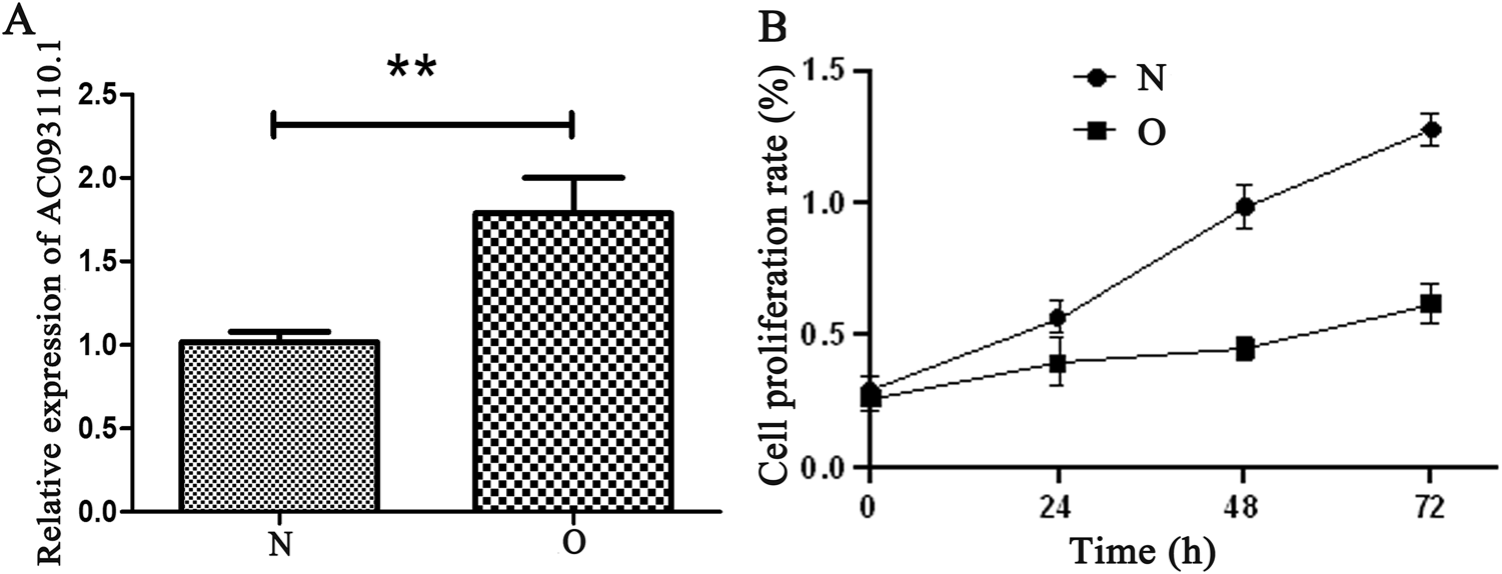
Expression and proliferation of MCF-7 cells after AC093110.1 overexpression. (**A**) Verification of relative expression of AC093110.1 after overexpression; (**B**) MTT detect the proliferation ability of MCF-7 cells after overexpression of AC093110.1. N and O represent normal cells and overexpressing AC093110.1 cells, respectively.

**Figure 11:**
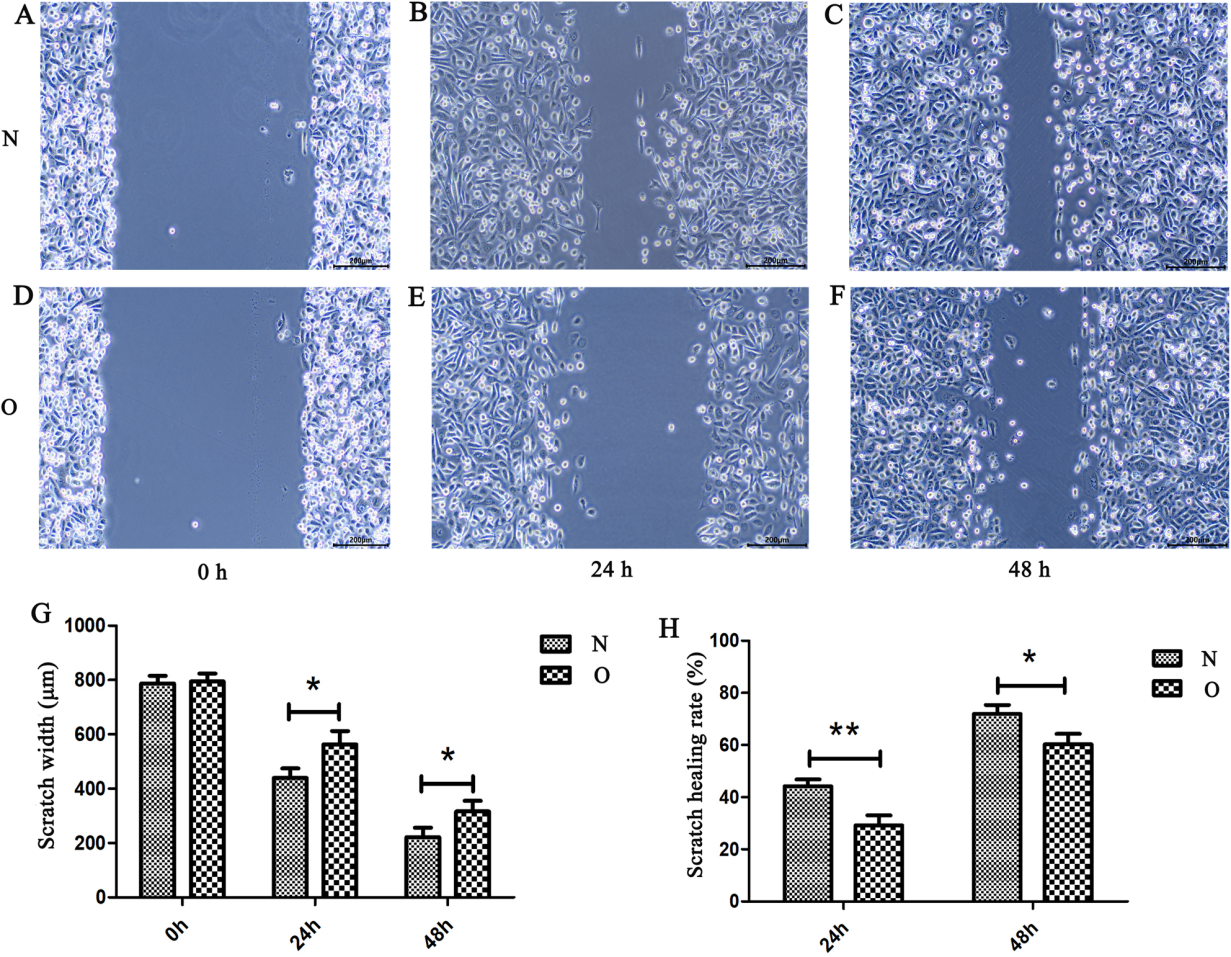
Migration of MCF-7 cells after AC093110.1 overexpression. (**A**) Cells migration capacity in normal MCF-7 at 0 h; (**B**) Cells migration capacity in normal MCF-7 cells at 24 h; (**C**) Cells migration capacity in normal MCF-7 cells at 48 h; (**D**) Cells migration capacity in AC093110.1 overexpressed cells at 0 h; (**E**) Cells migration capacity in AC093110.1 overexpressed cells at 24 h; (**F**) Cells migration capacity in AC093110.1 overexpressed cells at 48 h; (**G**) Scratch width of normal cells and overexpressed cells at different times. (**H**) Scratch healing rate of normal cells and overexpressed cells at different times. N and O represent normal cells and overexpressing AC093110.1 cells, respectively.

**Figure 12:**
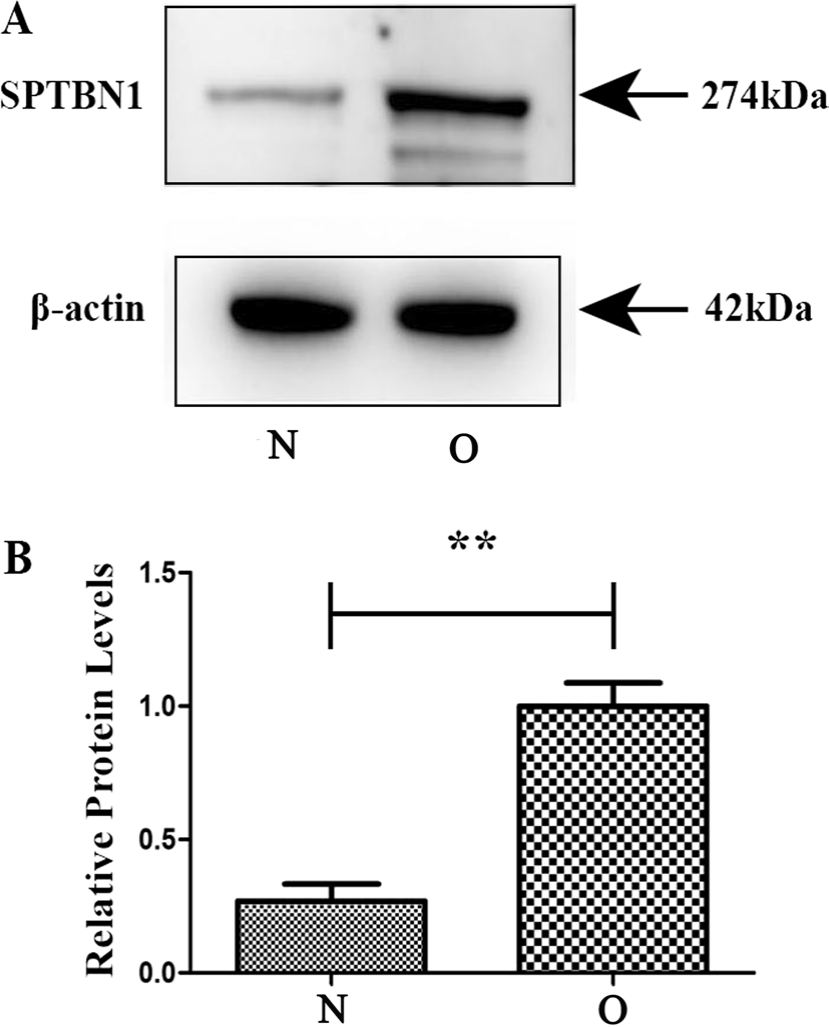
Western blotting assay (**A**) and quantitative analysis (**B**) of SPTBN1 after AC093110.1 overexpression in MCF-7 cells. N and O represent normal cells and overexpressing AC093110.1 cells, respectively. Usually the blots were cut prior to hybridisation with antibodies.

Bioinformatics analysis showed that AC093110.1 cis regulation of SPTBN1. Moreover, SPTBN1 and AC093110.1 have potential diagnostic and prognostic value, respectively. So we investigated the potential biological function of AC093110.1 at the cell level. AC093110.1 was overexpressed in human BC cell line MCF-7 (Fig. 10A). MTT detection showed that the proliferation rate in AC093110.1 overexpressed cells was significantly lower than that of normal MCF-7 cells (Fig. 10B). Conspicuously, overexpression of AC093110.1 inhibited cell migration (Fig. 11). Moreover, AC093110.1 cis regulated SPTBN1 in the bioinformatics analysis. Western blotting assay was used to detect the expression of SPTBN1 after AC093110.1 overexpression. The results showed that the expression level of SPTBN1 was up-regulated after AC093110.1 overexpression (Fig. 12). These results suggest that AC093110.1 may play an important role in BC cell proliferation and migration by regulating SPTBN1.

## Discussion

Previous studies based on TCGA data have revealed the diagnostic and prognostic value of lncRNAs, miRNAs and mRNAs for EBC^16^. In addition, the identification of abnormal methylation of genes helps the detection of BC^17^. However, we have not found integrated studies on DNA methylation with transcriptome data. Key genes screened out through the integration of DNA methylation with DEmRNA and DElncRNA have more research significance for the diagnosis and treatment of EBC. In this study, ALDH1A2, ALDH1L1, HSPB6, MME, MRGPRF, PENK, PITX1, SPTBN1, WDR86 and CAV2 were considered as potential diagnostic gene biomarkers in EBC. Furthermore, CAV2, MME, AC093110.1 and AC120498.6 were considered as potential prognosis gene biomarkers in EBC. In addition, AC093110.1 could regulate SPTBN1 expression and play an important role in cell proliferation and migration, which provides clues to clarify the regulatory mechanism of EBC.

Caveolin 2 (CAV2) is a member of the caveolin protein family, the down-regulation of CAV2 promote cell proliferation of HeLa epithelial cervical cancer and A549 lung adenocarcinoma cells^18^. Compared with normal tissue, CAV2 showed a downward trend in tumor tissue^19^. In the early detection of BC, CAV is considered as a new methylation marker^20^. Membrane metalloendopeptidase (MME) is a transmembrane glycoprotein that can degrade a variety of substrates^21^. Down-regulation of MME in tumor tissues of esophageal squamous cell carcinoma (ESCC) is associated with poor prognosis, late clinical stage and lymph node metastasis^21^. Overexpression of MME can inhibit substance P to promote the growth of cholangiocarcinoma^22^. In addition, MME hypermethylation has been found in BC, which may be the cause of reduced gene expression^23^. In this study, down-regulation of CAV2 and MME in EBC tumor tissues was associated with poor prognosis. This showed that CAV2 and MME may have high prognostic value in EBC.

Spectrin beta, non-erythrocytic 1 (SPTBN1) is also known as ELF. Inhibition of SPTBN1 can up-regulate the activity of transcriptional activator 3, thereby promoting the development of in hepatocellular carcinoma (HCC)^24^. Meanwhile, it is also a potential and reliable biomarker for predicting the prognosis of HCC patients^25^. Previous studies have found that reduced SPTBN1 expression is associated with worse prognosis of pancreatic cancer^26^. The knockdown of SPTBN1 enhances the migration and invasion potential of BC cells^27^. In this study, the differentially expressed genes, SPTBN1 was modified by DNA methylation. Moreover, AUC value was greater than 0.9 in the ROC curve. Therefore, we speculated that SPTBN1 might be potential diagnostic genes in EBC. In addition, bioinformatics analysis showed that AC093110.1 cis regulation of SPTBN1. AC093110.1 also has potential prognostic value. So we investigated the potential biological function of AC093110.1 at the cell level. The results clarify that AC093110.1 may play an important role in cell proliferation and migration by regulating the expression of SPTBN1, which provide clues for elucidating the regulation mechanism of EBC.

Aldehyde dehydrogenase 1 family member L1 (ALDH1L1) is a folate metabolizing enzyme with tumor suppressive properties^28^. In colon cancer, ALDH1L1 expression is decreased and mRNA levels are negatively correlated with promoter hypermethylation^29^. In HCC, compared with low expression of ALDH1L1, patients with high expression of ALDH1L1 have a significantly lower risk of recurrence and death^30^. In addition, ALDH1L1 is considered as a potential biomarker for poor prognosis of gastric cancer^31^. ALDH1L1 expression is inhibited in BC patients, and the mean hypermethylation level in the promoter region was positively correlated with the down-regulation of ALDH1L1^32^. In addition, high expression of ALDH1L1 is found to be associated with better overall survival in BC patients^33^. In this study, the differentially expressed genes, ALDH1L1 was modified by DNA methylation. Moreover, AUC value was greater than 0.9 in the ROC curve. Therefore, we speculated that ALDH1L1 might be potential diagnostic genes in EBC.

Aldehyde dehydrogenase 1 family member A2 (ALDH1A2) has been reported to be down-regulated in the early stages of human prostate cancer, and can be used as a candidate tumor suppressor gene for prostate cancer^34,35^. However, previous studies have found that high expression of ALDH1A2 mRNA is significantly associated with poorer survival in patients with non-small cell lung cancer (NSCLC)^36^. High expression of ALDH1A2 is found to be related to better overall survival in BC patients^33^. In this study, the expression of ALDH1A2 decreased in patients with EBC, suggesting that ALDH1A2 may play different regulatory roles in different cancers.

The hypermethylation and down-regulation of proenkephalin (PENK) in BC can lead to cell migration and adhesion defects^37^. The PENK may be a molecular marker of tumorigenesis of hormone-receptor positive (HR( +))/human epidermal growth factor receptor 2 negative (HER2(-)) in adolescents and young adults^38^. In this study, KEGG analysis showed that PENK participated in Neuroactive ligand-receptor interaction signal pathway. In addition, AUC value was 0.907 in ROC curve. This further showed that PENK could play an important role in EBC.

Heat shock protein family B (small) member 6 (HSPB6), also known as Hsp20, and its expression level is negatively correlated with the degree of malignancy in ovarian cancer^39^. Decreased expression of HSP20 is associated with TNM stage, lymph node metastasis, and tumor recurrence, and may be valuable as a prognostic tumor marker^40^. Compared with normal lung tissue, the expression of paired like homeodomain 1 (PITX1) is up-regulated in NSCLC and is associated with a poorer prognosis^41^. PITX1 has been reported to be significantly increased in different histological classifications of BC, and is positively correlated with metastatic relapse-free survival and distant metastasis-free survival^43,44^. In this study, HSPB6 and PITX1 were down-regulated and up-regulated in EBC, respectively. This is consistent with the expression in other cancers. Therefore, it is speculated that HSPB6 and PITX1 could play an important regulatory role in the physiological and pathological process of EBC.

There are few studies on WD repeat domain 86 (WDR86) and MAS related GPR family member F (MRGPRF) in cancer. However, they are found to be significantly related to diagnosis of EBC in this study, which implies that they play an important regulatory role in the development of EBC.

In this study, analysis of EBC in TCGA revealed methylated modification status of ALDH1A2, ALDH1L1, HSPB6, MME, MRGPRF, PENK, PITX1, SPTBN1, WDR86 and CAV2, and clarified that they may be potential diagnostic gene biomarkers. In order to further prove abnormal methylation modification in EBC, we verified the methylation modification status of ALDH1A2, ALDH1L1, HSPB6, MME, MRGPRF, PENK, PITX1, SPTBN1, WDR86 and CAV2 in the GSE32393 dataset. The GSE32393 dataset contains methylation modification data from EBC tissue samples, which is consistent with TCGA^45^. The results showed that the methylation modification state was consistent with the result in TCGA. Genes methylation patterns are related to gene expression regulation^46,47^. These results suggest that abnormal methylation of genes plays an important role in the progression of EBC.

In addition to the above 10 genes, we also found that many genes had important regulatory roles in EBC, such as four and a half LIM domains 1 (FHL1), caveolin 1 (CAV1), long intergenic non-protein coding RNA 1614 (LINC01614), FOXD3 antisense RNA 1 (FOXD3-AS1) etc. FHL1 is a tumor suppressor gene, which is down-regulated in BC^48^. CAV1 is down-regulated in BC cells and tissues, and it is revealed that CAV1 plays an essential regulatory role in BC by regulating lysosomal function and autophagy^49^. LINC01614 is highly expressed in BC and has strong prognostic value, which can be used as a potential biomarker to predict the prognosis of BC^50^. Compared with normal tissues, FOXD3-AS1 has a significantly higher expression in BC tissues. Moreover, patients with low FOXD3-AS1 expression have a higher survival rate, smaller tumor size and fewer distant metastases^51^. In this study, we found that FHL1, CAV1 were down-regulated and LINC01614, FOXD3-AS1 were up-regulated in EBC. The expression trend was consistent with previous studies, which further suggests that they may play an important regulatory role in EBC.

Certain limitations exist in this study. First of all, the RT-PCR verification sample size is too small, which leads to errors in the verification results and bioinformatics analysis. The sample needs to be expanded for further verification. Secondly, the molecular mechanisms of the identified key genes were still unclear and require further study in EBC. Thirdly, the role of the identified genes in each subtype of BC needs to be further studied.

## Conclusion

In this study, these results indicate that identified genes may be used as potential clinical biomarkers of EBC. Among them, ALDH1A2, ALDH1L1, HSPB6, MME, MRGPRF, PENK, PITX1, SPTBN1, WDR86 and CAV2 may be considered as the potential diagnostic gene biomarkers for EBC. Strikingly, CAV2, MME, AC093110.1 and AC120498.6 were significantly actively correlated with survival. In addition, AC093110.1 could regulate SPTBN1 expression and play an important role in cell proliferation and migration, which provides clues to clarify the regulatory mechanism of EBC. In short, the identification of these genes can help in the early diagnosis and treatment of EBC.

## Materials and methods

### Datasets

The data of mRNA (read counts data), lncRNA (read counts data) and DNA methylation (DNA methylation chip data) were downloaded from TCGA (https://tcga-data.nci.nih.gov/tcga/) on June 30, 2020. A total of 1098 patients with BC were included in the dataset, including 1098 patients with clinical data, 1092 patients with RNA-seq data and 1095 patients with methylation array data. According to the clinical information stage, 804 patients with EBC (stage I-II) were selected. Finally, selected patients all had mRNA data, lncRNA data and DNA methylation data. Detailed data was shown in Table 6. We used the R software (version 3.5.1; Bell Laboratories, formerly AT&T, now Lucent Technologies, Murray Hill, NJ, USA) to analyze mRNA, lncRNA and DNA methylation data.

**Table 6:** Transcriptome mRNA, lncRNA and DNA methylation data of early breast cancer patients.

Data type	Paracancerous	Early breast cancer	Data type
mRNA	61	560	Read counts data
lncRNA	61	560	Read counts data
DNA methylation	61	560	DNA methylation chip data

61 and 560 represent the number of paracancer and early breast cancer samples, respectively.

### Differential analysis of mRNAs and lncRNAs

The DEmRNAs and DElncRNAs were evaluated in the R-bioconductor package DESeq^52^. Firstly, the RNAs read counts = 0 distributed greater than 20% in the case sample or the RNAs read counts = 0 distributed greater than 20% in the normal sample were deleted. Then, false discovery rate (FDR) < 0.05 and absolute value of log2FoldChange > 2 were used to identify DEmRNAs and DElncRNAs. Log2FoldChange > 2 and log2FoldChange < -2 represented up-regulation and down-regulation, respectively.

### Biological function enrichment analysis of DEmRNAs

In order to explore the biological function of DEmRNAs, GO (including biological process, molecular function and cellular component) and KEGG functional enrichment analysis of DEmRNAs were performed by using clusterProfiler package in R^53^. The detailed technical parameters of enrichment were organism = has, pvalueCutoff = 0.05, qvalueCutoff = 0.1. FDR < 0.05 was considered statistically significant.

### DNA methylation analysis

The COHCAP package in R was used to screen DMSs^54^. The Δβ was the difference of methylation site expression values between case and normal. In order to ensure the reliability of difference analysis results, 485,577 methylation sites detected were preprocessed. Filter out the methylated sites whose expression value β is not available (NA) and the distribution is greater than 20% in the sample. The COHCAP.annotate function was used to annotate the methylation sites and remove the methylation sites on the sex chromosomes. After preprocess, we perform PCA of the remaining methylation sites. Subsequently, β > 0.7 or β < 0.3 were selected for methylation sites. Finally, Δβ > 0.2 and FDR < 0.05 were used to screen DMSs and differentially methylation regions (DMRs).

### Correlation analysis of DNA methylation with DEmRNAs

Chromosomal location information of DEmRNAs and DElncRNAs was downloaded from ensemble database. Then, mRNA-lncRNA distance information was extracted with bedtools intersect tool^55^. DEmRNAs were searched in the upstream and downstream of DElncRNAs within the range of 100 kilobase (kb). In order to obtain DEmRNAs that were jointly regulated by lncRNA and related with DNA methylation, we took the intersection of lncRNA cis regulated mRNA and mRNAs corresponding to the DMSs. P < 0.05 and absolute value of cor > 0.2 were used to perform Pearson correlation analysis on DMSs and DEmRNAs. In addition, the relationship between DEmRNAs and differential methylated on CpG island was integrated. Finally, Venn analysis (http://www.bioinformatics.com.cn/) was used to show the DEmRNAs obtained from the above three steps.

### Methylation verification, diagnostic and prognostic analysis of identified DEmRNAs and DElncRNAs

In order to evaluate the potential diagnostic utility and prognostic value of the identified genes in EBC, diagnostic analysis and prognostic analysis were performed in the TCGA database. The pROC package in R was used for diagnostic analysis. The sensitivity and specificity at the cut-offs were determined referring to previous report^56^. The diagnostic ability was evaluated by the AUC values in the ROC curve. Survival and Survminer software packages in R were used to for survival analysis and survival curve drawing. The prognostic ability was evaluated by survival curve. Subsequently, the GSE32393 dataset was downloaded from the Gene Expression Omnibus (GEO, https://www.ncbi.nlm.nih.gov/geo/) database. Methylation modification state verification of the identified genes was performed in the GSE32393 dataset (Normal:Case = 23:100).

### RT-PCR validation of identified genes

6 patients with clinical stage I-II EBC were enrolled. The EBC tissue samples (disease group) and adjacent normal tissues samples (control group) were obtained for RT-PCR. The TRIzol kit was used for extracted total RNA. Then, FastKing cDNA first chain synthesis kit (KR116, TIANGEN) was used for mRNA reverse transcription. RT-PCR was performed using SuperReal PreMix Plus (SYBR Green) (FP205, TIANGEN). Glyceraldehyde-3-phosphate dehydrogenase (GAPDH) and actin beta (ACTB) were used as internal reference. The relative gene expression levels were calculated using the 2^-ΔΔCt^ method^57^. This study was approved by the ethics committee the Shijiazhuang people’s Hospital (202060). The written consent was obtained from the all patients.

### Validation at the cell level

Bioinformatics analysis showed that AC093110.1 cis regulation of SPTBN1. Moreover, SPTBN1 and AC093110.1 have potential diagnostic and prognostic value, respectively. So we investigated the potential biological function of AC093110.1 at the cell level. AC093110.1 was overexpressed in human BC cell line MCF-7. The MCF-7 cells were cleaned with phosphate buffered saline (PBS) before transfection, and then 3 × 10^5^ cells were inoculated into each well of the 6-well plate with 1.5 ml low serum minimum essential medium (MEM). Subsequently, the cells were incubated in 37 ˚C incubator. AC093110.1 was added to 250 μl MEM, and then mixed with 250ul MEM containing 5ul lipofectamine 2000 transfection reagent. The mixture was incubated at room temperature for 20 min. Add the mixture to the cultured 6-well plate. The cell transfection efficiency was detected after 48 h. The expression level of AC093110.1 was detected by RT-PCR. The MTT assay was used to analyze the affect of AC093110.1 on cell proliferation. Cell wound scratch assay was used to analyze the affect of AC093110.1 on cell migration. In addition, after overexpression of AC093110.1, western blot was performed to detect the expression of SPTBN1 adjacent to AC093110.1. Usually the blots were cut prior to hybridisation with antibodies.

### Statistical analysis

GraphPad Prism was used to perform all the data statistics. For the RT‑qPCR experiments, one‑way ANOVA and Duncan's multiple range test was used to assess differences between case and normal groups. Results were presented as the mean ± SD. P < 0.05 was considered as statistical significance. Experiments were repeated independently at least 3 times.

### Ethics approval and consent to participate

This study was approved by the ethics committee the Shijiazhuang people’s Hospital (202060). The written informed consent was obtained from the all patients. All participants were informed as to the purpose of this study, and that this study complied with the Declaration of Helsinki.

## Supplementary Information

Supplementary Information 1.

Supplementary Information 2.

Supplementary Information 3.

Supplementary Information 4.

Supplementary Information 5.

## Data Availability

All data generated or analyzed during this study are included in this published article.
