# Author Correction: Epigenetic study of early breast cancer (EBC) based on DNA methylation and gene integration analysis

**DOI:** 10.1038/s41598-022-13331-w

**Published:** 2022-05-26

**Authors:** Wenshan Zhang, Haoqi Wang, Yixin Qi, Sainan Li, Cuizhi Geng

**Affiliations:** 1 Department of Breast Center, The Fourth Hospital of Hebei Medical University, 169 Tianshan Street, Shijiazhuang, 050011 Hebei People’s Republic of China; 2 Gland Surgery, Shijiazhuang People’s Hospital, Shijiazhuang, People’s Republic of China

Correction to: *Scientific Reports* 10.1038/s41598-022-05486-3, published online 07 February 2022

In the original version of this Article, Affiliation 1 and 2 were not listed in the correct order. The correct affiliations are listed below:

Affiliation 1:

Department of Breast Center, The Fourth Hospital of Hebei Medical University, 169 Tianshan Street, Shijiazhuang, Hebei 050011, People’s Republic of China

Affiliation 2:

Gland Surgery, Shijiazhuang People’s Hospital, Shijiazhuang, People’s Republic of China

Furthermore, Affiliation 1 was omitted for Wenshan Zhang.

The original Article has been corrected.

