# Supplementary material for: Epigenetic study of early breast cancer (EBC) based on DNA methylation and gene integration analysis
Source: Sci Rep. 2022 Feb 7;12:1989. doi: 10.1038/s41598-022-05486-3 (PMC8821628; doi:10.1038/s41598-022-05486-3)
Supplement: Supplementary file 1 — Supplementary Information 1. [file 41598_2022_5486_MOESM1_ESM.docx]

**Western blot original blot**


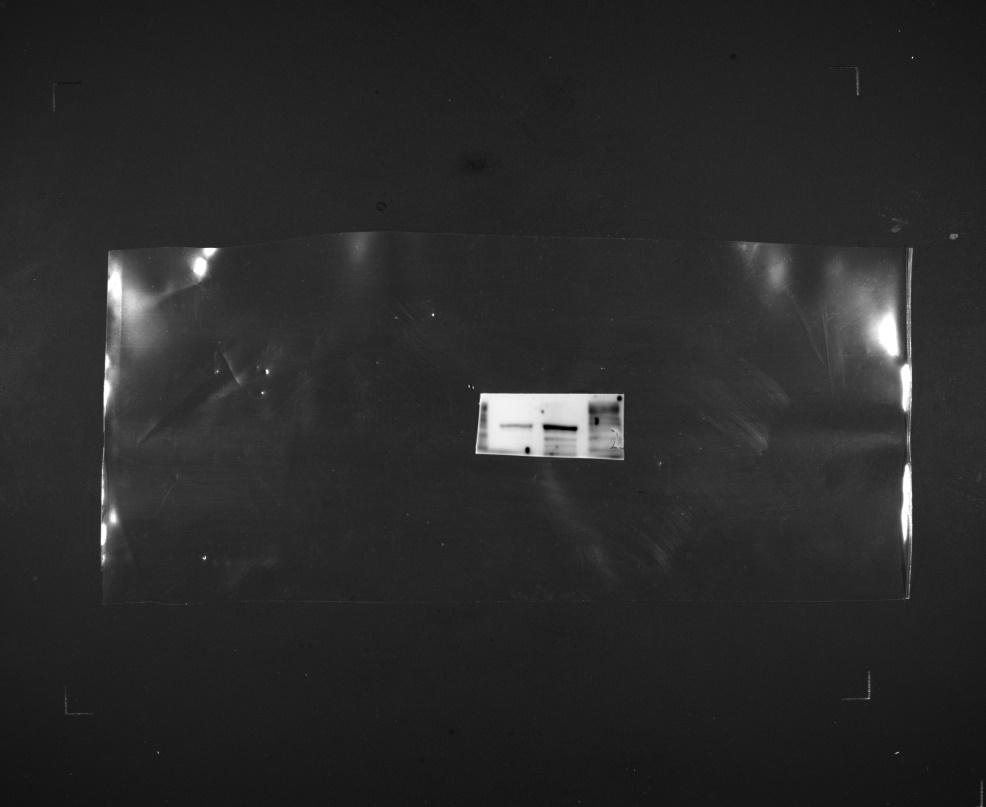


The first time: Western blotting assay of SPTBN1 after AC093110.1 overexpression in MCF-7 cells.


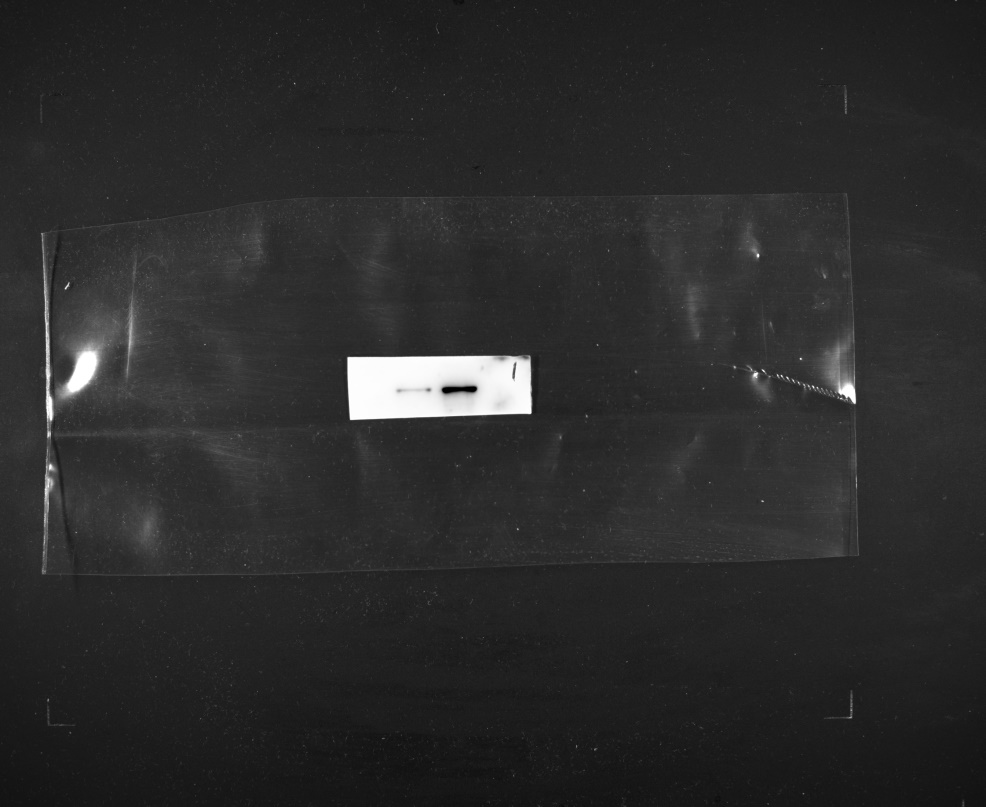


The second time: Western blotting assay of SPTBN1 after AC093110.1 overexpression in MCF-7 cells.


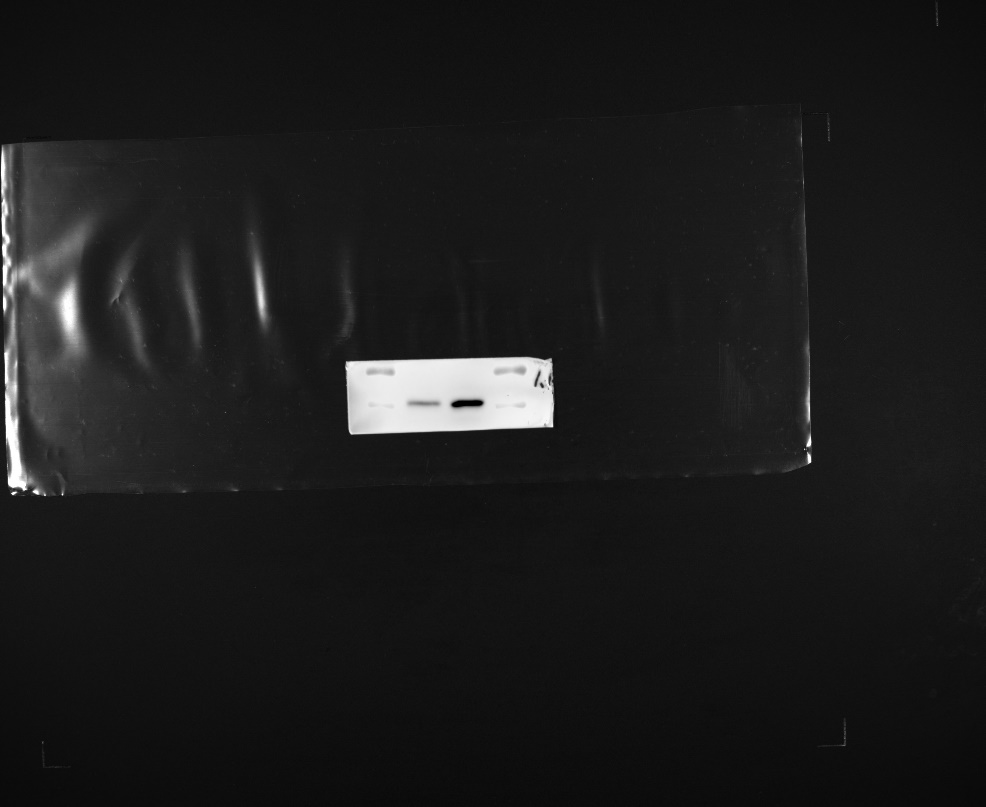


300kDa

The third time: Western blotting assay of SPTBN1 after AC093110.1 overexpression in MCF-7 cells.
