# Supplementary material for: Epigenetic study of early breast cancer (EBC) based on DNA methylation and gene integration analysis
Source: Sci Rep. 2022 Feb 7;12:1989. doi: 10.1038/s41598-022-05486-3 (PMC8821628; doi:10.1038/s41598-022-05486-3)
Supplement: Supplementary file 2 — Supplementary Information 2. [file 41598_2022_5486_MOESM2_ESM.docx]

**Supplementary data**

**Supplementary Figure 1: Heat map of top 100 DEmRNAs (A) and DElncRNA (B).**

Complete‑linkage method combined with Euclidean distance is used to construct clustering. Each row represents a DEmRNAs or DElncRNA, and each column represents a sample. DEmRNAs or DElncRNA clustering tree is shown on the left. Red indicates above the reference channel (high expression genes). Green indicates below the reference channel (low expression genes).

**Table S1 DMSs-DEmRNAs correlation analysis**

**Table S2 Relationship between differentially methylated regions and differentially expressed genes**
