# Supplementary figures and images for: Epigenetic study of early breast cancer (EBC) based on DNA methylation and gene integration analysis
Source: Sci Rep. 2022 Feb 7;12:1989. doi: 10.1038/s41598-022-05486-3 (PMC8821628; doi:10.1038/s41598-022-05486-3)

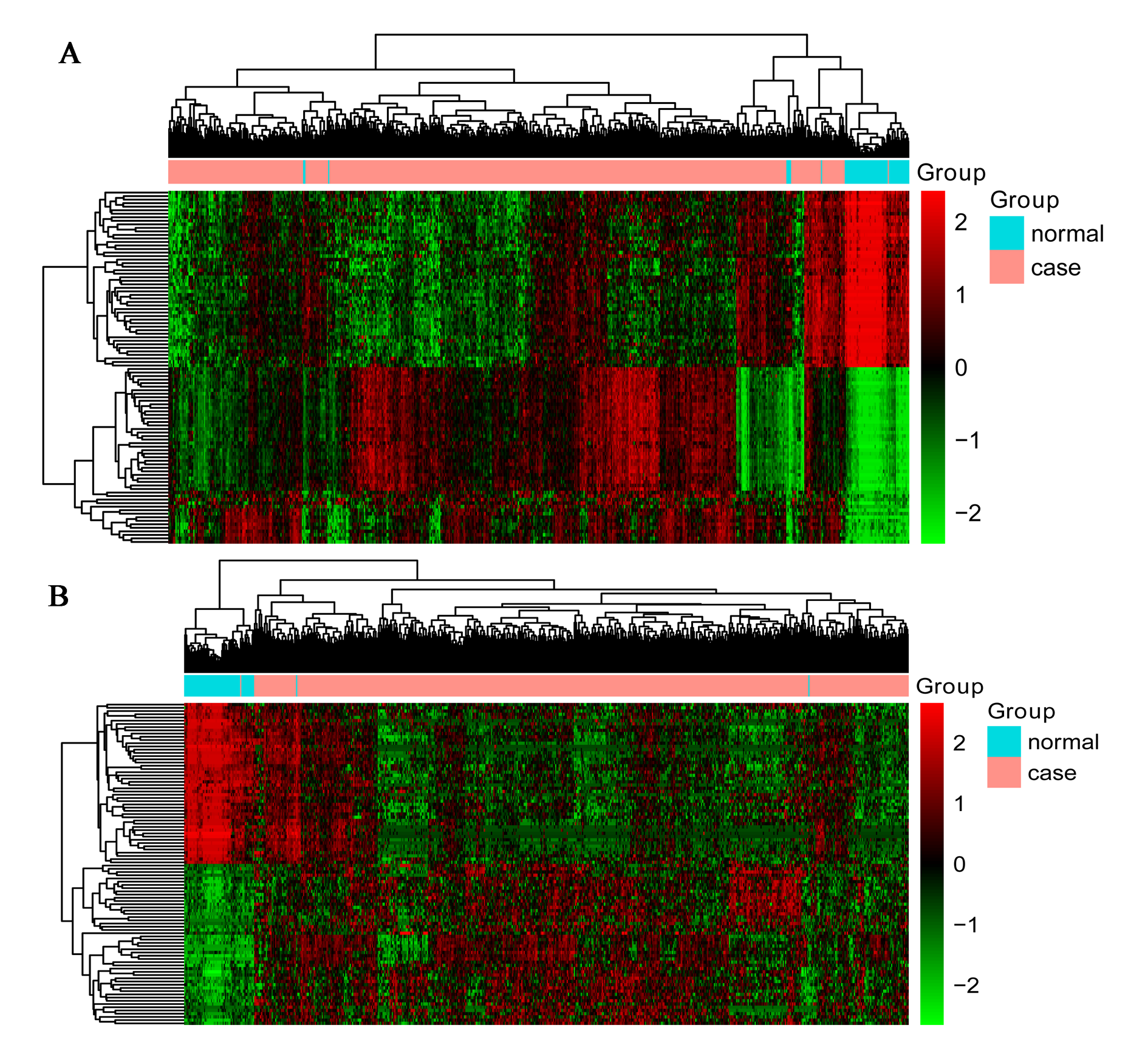

Supplement: Supplementary file 3 — Supplementary Information 3. [file 41598_2022_5486_MOESM3_ESM.tif]
