# Supplementary material for: Epigenetic study of early breast cancer (EBC) based on DNA methylation and gene integration analysis
Source: Sci Rep. 2022 Feb 7;12:1989. doi: 10.1038/s41598-022-05486-3 (PMC8821628; doi:10.1038/s41598-022-05486-3)
Supplement: Supplementary file 5 — Supplementary Information 5. [file 41598_2022_5486_MOESM5_ESM.docx]

**Table S2 Relationship between differentially methylated regions and differentially expressed genes**

| **Cpg.island** | **Island.delta.beta** | **Island.fdr** | **Methylation** | **Genes** | **Log2FoldChange** | **Padj** | **Up/Down** |
| --- | --- | --- | --- | --- | --- | --- | --- |
| chr1:119529819-119530712 | 0.241660423 | 4.95922E-06 | Increased | TBX15 | -2.136787485 | 3.3E-45 | Down |
| chr1:65991001-65991811 | 0.241660423 | 0.005891716 | Increased | LEPR | -2.97054099 | 1.2E-81 | Down |
| chr1:78511577-78512161 | 0.241660423 | 0.000450316 | Increased | GIPC2 | -2.655998996 | 3.22E-51 | Down |
| chr10:100993820-100994188 | 0.241660423 | 2.73428E-05 | Increased | HPSE2 | -3.15353547 | 1.32E-28 | Down |
| chr11:105481126-105481422 | 0.241660423 | 2.39821E-07 | Increased | GRIA4 | -3.191411231 | 6.48E-25 | Down |
| chr11:110581839-110583883 | 0.241660423 | 0.034548546 | Increased | ARHGAP20 | -3.073489615 | 4.6E-83 | Down |
| chr11:30038522-30038823 | 0.241660423 | 7.50758E-07 | Increased | KCNA4 | -3.691972653 | 1.37E-19 | Down |
| chr11:44332314-44332757 | 0.241660423 | 0.032608265 | Increased | ALX4 | -2.353455944 | 4.25E-18 | Down |
| chr12:128751041-128753151 | 0.241660423 | 0.017961007 | Increased | TMEM132C | -4.878111704 | 8.91E-58 | Down |
| chr12:95941906-95942979 | 0.241660423 | 0.000131939 | Increased | USP44 | -2.326384709 | 2.34E-39 | Down |
| chr13:33589928-33591428 | 0.241660423 | 0.024433407 | Increased | KL | -2.466903884 | 1.91E-43 | Down |
| chr13:78492425-78493382 | 0.241660423 | 1.64371E-07 | Increased | EDNRB | -2.219947582 | 1.17E-48 | Down |
| chr14:38678245-38680937 | 0.241660423 | 0.0018732 | Increased | SSTR1 | -2.60890219 | 1.63E-29 | Down |
| chr15:58357309-58358776 | 0.241660423 | 0.001440772 | Increased | ALDH1A2 | -2.85539513 | 5.39E-47 | Down |
| chr18:74961556-74963822 | 0.241660423 | 1.55742E-09 | Increased | GALR1 | -2.429976425 | 2.66E-14 | Down |
| chr19:36246328-36247982 | 0.241660423 | 7.98974E-06 | Increased | HSPB6 | -4.569373129 | 8.37E-99 | Down |
| chr19:38746638-38747379 | 0.241660423 | 0.002561723 | Increased | PPP1R14A | -2.205092904 | 6.07E-25 | Down |
| chr2:106681982-106682403 | 0.241660423 | 0.016781487 | Increased | C2orf40 | -2.78317115 | 4.32E-19 | Down |
| chr2:127413696-127414171 | 0.241660423 | 2.14986E-05 | Increased | GYPC | -2.03853436 | 1.54E-53 | Down |
| chr2:220283200-220283750 | 0.241660423 | 0.018844836 | Increased | DES | -2.149638945 | 6.15E-10 | Down |
| chr2:229045957-229046553 | 0.241660423 | 0.010410987 | Increased | SPHKAP | -2.759937997 | 8.58E-10 | Down |
| chr2:96990857-96991283 | 0.241660423 | 2.14986E-05 | Increased | ITPRIPL1 | -2.334447457 | 1.16E-43 | Down |
| chr20:57875317-57876126 | 0.241660423 | 0.001572109 | Increased | EDN3 | -2.239647133 | 9.61E-08 | Down |
| chr3:12045652-12046627 | 0.241660423 | 0.002151107 | Increased | SYN2 | -2.784255042 | 1.88E-36 | Down |
| chr3:133464949-133465420 | 0.241660423 | 0.021484943 | Increased | TF | -2.244639334 | 9.3E-13 | Down |
| chr3:238391-240140 | 0.241660423 | 0.03590371 | Increased | CHL1 | -3.017414228 | 1.11E-41 | Down |
| chr3:62357639-62359774 | 0.241660423 | 0.032832911 | Increased | FEZF2 | -2.183188012 | 0.000712883 | Down |
| chr4:42399152-42400802 | 0.241660423 | 0.002437653 | Increased | SHISA3 | -2.614676261 | 2.61E-26 | Down |
| chr5:115151348-115152713 | 0.241660423 | 9.42966E-07 | Increased | CDO1 | -3.780411804 | 1.54E-73 | Down |
| chr6:28226979-28227483 | 0.241660423 | 0.008264256 | Increased | NKAPL | -2.708323834 | 3.9E-81 | Down |
| chr6:6007387-6007797 | 0.241660423 | 0.006917427 | Increased | NRN1 | -2.314402594 | 3.7E-47 | Down |
| chr7:116139774-116140352 | 0.241660423 | 0.000847964 | Increased | CAV2 | -2.853949712 | 9.19E-95 | Down |
| chr7:127880750-127881375 | 0.241660423 | 0.008055999 | Increased | LEP | -6.077687514 | 7.16E-65 | Down |
| chr7:153583317-153585666 | 0.241660423 | 8.62475E-08 | Increased | DPP6 | -2.528817833 | 6.57E-15 | Down |
| chr7:153748407-153750444 | 0.241660423 | 0.001683771 | Increased | DPP6 | -2.528817833 | 6.57E-15 | Down |
| chr7:27169572-27170638 | 0.241660423 | 8.52118E-07 | Increased | HOXA4 | -2.31041962 | 6.1E-42 | Down |
| chr7:27182613-27185562 | 0.241660423 | 0.020597103 | Increased | HOXA5 | -2.540698367 | 4.23E-41 | Down |
| chr8:25900562-25905842 | 0.241660423 | 0.044438789 | Increased | EBF2 | -2.327252608 | 1.58E-46 | Down |
| chr8:37822486-37824008 | 0.241660423 | 0.022750622 | Increased | ADRB3 | -2.76030416 | 1.72E-19 | Down |
| chr8:55370170-55372525 | 0.241660423 | 1.09261E-06 | Increased | SOX17 | -2.245005498 | 1.66E-49 | Down |
| chr8:57358126-57359415 | 0.241660423 | 0.001284936 | Increased | PENK | -4.015868989 | 1.09E-31 | Down |
| chr8:86350765-86351196 | 0.241660423 | 8.85546E-05 | Increased | CA3 | -3.786703672 | 5.18E-45 | Down |
